# Exposure to blue light during lunch break: effects on autonomic arousal and behavioral alertness

**DOI:** 10.1186/s40101-017-0148-4

**Published:** 2017-07-11

**Authors:** Emi Yuda, Hiroki Ogasawara, Yutaka Yoshida, Junichiro Hayano

**Affiliations:** Department of Medical Education, Nagoya City University Graduate School of Medical Sciences, 1 Kawasumi Mizuho-cho Mizuho-ku, Nagoya, 467-8601 Aichi Japan

**Keywords:** Alertness, Arousal, Blue light, Heart rate variability, Melanopsin, Non-image forming vision, Orange light, Organic light-emitting diode, Psychomotor vigilance

## Abstract

**Background:**

Exposures to melanopsin-stimulating (melanopic) component-rich blue light enhance arousal level. We examined their effects in office workers.

**Main body of abstract:**

Eight healthy university office workers were exposed to blue and orange lights for 30 min during lunch break on different days. We compared the effects of light color on autonomic arousal level assessed by heart rate variability (HRV) and behavioral alertness by psychomotor vigilance tests (PVT). Heart rate was higher and high-frequency (HF, 0.150.45 Hz) power of HRV was lower during exposure to the blue light than to orange light. No significant difference with light color was observed, however, in any HRV indices during PVT or in PVT performance after light exposure.

**Short conclusion:**

Exposure to blue light during lunch break, compared with that to orange light, enhances autonomic arousal during exposure, but has no sustained effect on autonomic arousal or behavioral alertness after exposure.

## Background

Along with the development of lighting elements capable of various color-rendering, the introduction of color lighting in life environment is taking on reality. Such lightings may be used for adjusting our mind and physical states to suit our desired activities, such as work, study, rest, and sleep [1–4]. In previous studies [5, 6], we have demonstrated that exposure to melanopsin-stimulating (melanopic) component-rich blue light enhances autonomic arousal and behavioral alertness. These observations, however, were obtained in laboratory settings. In this study, we examined the effects of exposure to blue light or orange lights during the lunch break on autonomic arousal and behavioral alertness in office workers.

## Main text

### Subjects and protocols

We studied eight healthy university office workers (mean age ± SD, 27 ± 7 years, range 21–39 years, 2 females) with normal color vision who were not taking any medications for >2 weeks and displayed a normal sinus rhythm on electrocardiogram (ECG).

**Fig. 1:**
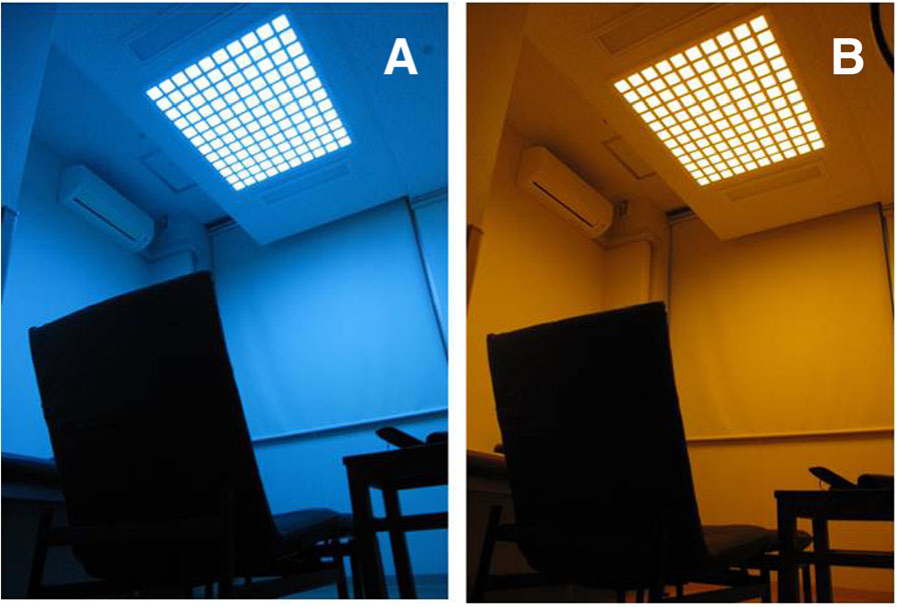
Ceiling light chamber with organic light-emitting diode (OLED) when the lighting is **a** blue and **b** orange

We compared the effects on heart rate variability (HRV) and psychomotor vigilance test (PVT) caused by exposures to blue and orange lights during the lunch break in workplace. We prepared a light chamber in the workplace with an organic light-emitting diode (OLED) ceiling light system with adjustable color and brightness (Fig. 1), whose details have been reported elsewhere [6]. Table 1 shows the optical characteristics of blue and orange lights.

**Table 1:** Characteristics of organic light-emitting diode (OLED) lights used in this study

	Blue	Orange
Illuminance, lx	12.9	17.7
Irradiance, μW/cm^2^	8.02	6.54
Chromaticity (*x*, *y*)	0.14, 0.16	0.56, 0.42
Weighted mean wavelength, nm	485	622
PFD, μmol/m^2^/s	0.323	0.333
Melanopic PFD, μmol/m^2^/s^a^	0.225	0.026
Relative melanopic content, %	69.6	7.9

*PFD* photon flux density

^a^Calculated from melanoptic spectral efficiency adjusted for human pre-receptoral filtering [13, 14]

Experiments with the two light colors were performed on separated days with an interval of 5–7 days for washout. The color orders were counterbalanced across subjects. On each experimental day, Holter ECG was recorded continuously during experiment. Subjects took a light lunch (the same menu on two experimental days) with a drink not containing caffeine or alcohol at the university cafeteria. During the last 30 min of lunch break, they were exposed to blue or orange light in the light chamber (air-conditioned at 24 ± 2 °C), sitting in a low chair with 60° backrest just below the ceiling light.

After the lunch break, a 5-min PVT was performed in their work office which was illuminated by fluorescent lamps with a correlated color temperature of 4010 K, an illuminance of 450 lx, a photon flux density of 6.7 μmol/m^2^/s, and a relative melanopic component of 20.4%. PVT was performed with validated software (PC-PVT) [7]. The detailed method for PVT has been reported elsewhere [6]. The anticipation was set at 100 ms, deadline at 65,000 ms, minor lapse at 500 ms, and total trial time at 300 s.

### Data analysis

R-R interval time series obtained from Holter ECG were extracted for the durations of OLED light exposure (30 min) and PVT (5 min) for the analysis of HRV. HRV was analyzed by the methods reported elsewhere [8, 9]. The power of the low-frequency (LF, 0.04–0.15 Hz) and the high-frequency (HF, 0.15–0.40 Hz) components were computed by fast Fourier transformation. We used heart rate as an index of sympatho-vagal balance, HF power as cardiac vagal function, and LF-to-HF power ratio (LF/HF) as sympathetic predominance in cardiac autonomic functions. The power of LF and HF components was transformed into natural logarithmic value.

PVT performance was analyzed according to the earlier studies [10]. We measured the number of minor lapses (reaction time (RT) ≥500 ms) with transformation ($\sqrt{x}+\sqrt{x+1}$), fastest and slowest 10th percentile RTs, and difference between fastest and slowest RTs.

We used the Statistical Analyses System version 9.4 (SAS institute Inc., Cary, NC, USA) for statistical analysis. To examine the effects of light color on HRV indices and PVT performance, we compared the values between blue and orange lights with paired *t* test. *P* < 0.05 was considered to be statistically significant.

## Results

**Fig. 2:**
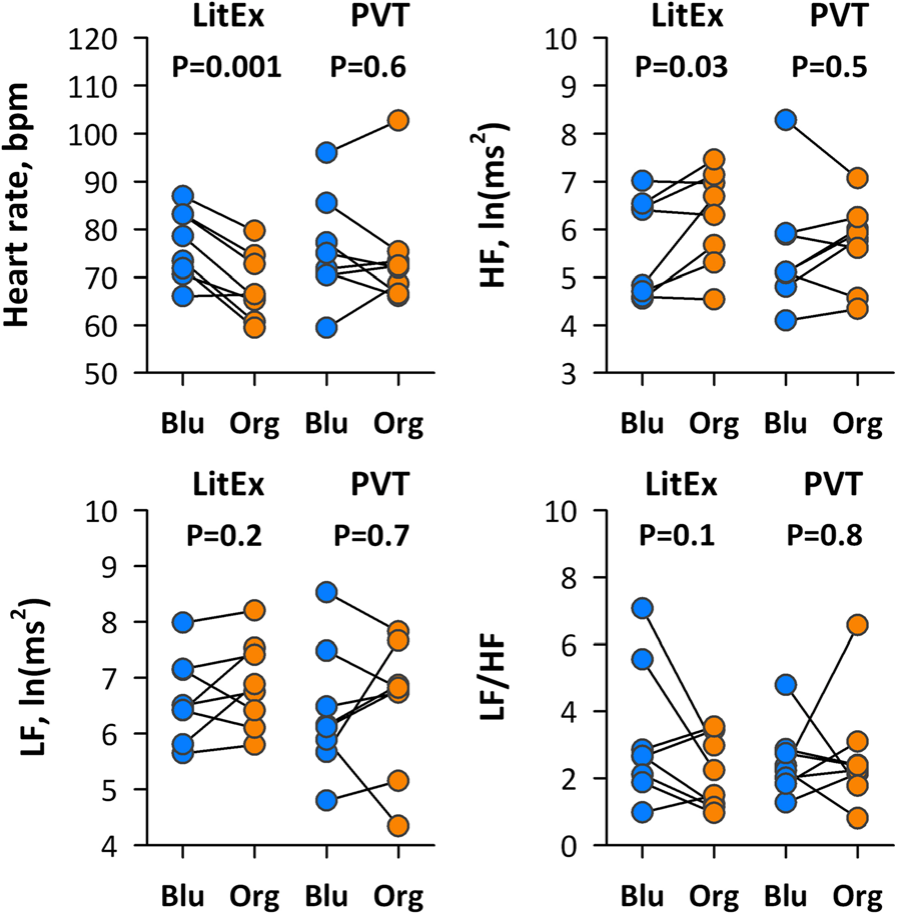
Differences between blue and orange lights in heart rate and heart rate variability (HRV) indices during light exposure (LitEx) and psychomotor vigilance test (PVT) after exposure in individual subjects (*n* = 8). *Blu* blue light, *HF* high-frequency component, *LF* low-frequency component, *LF*/*HF* LF-to-HF ratio in power, *Org* orange light

**Fig. 3:**
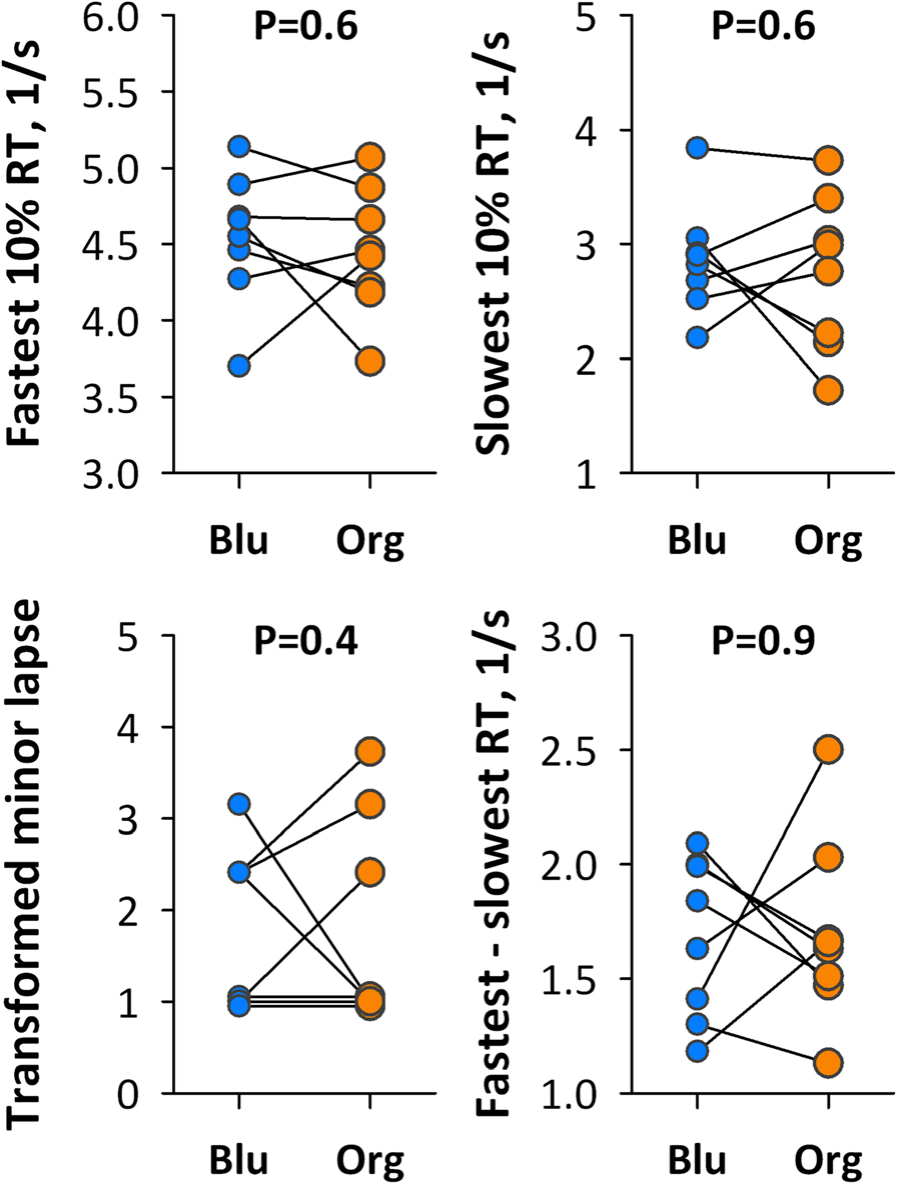
Differences between blue and orange lights in the performance of PVT after light exposure in individual subjects (*n* = 8). *Blu* blue light, *Org* orange light, *RT* reaction time

The HRV indices during exposures to blue and orange lights and during PVT after exposure in individual subjects are shown in Fig. 2. Heart rate was higher (*P* = 0.001) and HF power was lower (*P* = 0.03) during exposure to blue light than to orange light, whereas there was no significant difference with light color in any HRV indices during PVT after exposure. As shown in Fig. 3, no significant difference with light color was observed in any indices of PVT performance after exposure.

## Conclusions

From these findings, we conclude that compared with orange light, blue light during lunch break enhances autonomic arousal during exposure, but causes no sustained differences in autonomic arousal or behavioral alertness after exposure.

In this study, we observed higher heart rate and lower HF power during exposure to blue light than orange light. These observations seem consistent with earlier studies suggesting autonomic arousal effects of melanopic component-rich blue light [1–6]. In contrast, we failed to detect significant difference with light color in autonomic indices during PVT or PVT performed after exposure. This seems inconsistent with those of an earlier study of Chellappa et al. [2] and of our previous study [6]. We performed PVT 30 min after light exposure in this study, while it was performed during light exposure in Chellappa’s study [2] and <5 min after exposure in our previous study [6]. The difference may be attributable to the duration of effects. In any case, however, exposure to blue light during lunch break may not cause substantial difference from orange light in behavioral alertness during afternoon’s work.

This study has several limitations. First, this study was performed in university office workers. Our observations may not be generalized for other types of workers. Second, the intensity of blue light we used was low (PFD, 0.323 μmol/m^2^/s), but the effects may differ depending on light intensity [11, 12]. Third, because we did not measure melatonin secretion, we were unable to determine whether the light exposures affect the entrainment to environmental light–dark cycles or not.

## Abbreviations

ECG: Electrocardiogram
HF: High frequency
HRV: Heart rate variability
LF: Low frequency
LF/HF: LF-to-HF ratio
OLED: Organic light-emitting diode
PFD: Photon flux density
PVT: Psychomotor vigilance test
RT: Reaction time
